# The Subcellular Distribution of T-Type Ca^2+^ Channels in Interneurons of the Lateral Geniculate Nucleus

**DOI:** 10.1371/journal.pone.0107780

**Published:** 2014-09-30

**Authors:** Vaneeda Allken, Joy-Loi Chepkoech, Gaute T. Einevoll, Geir Halnes

**Affiliations:** 1 Dept. of Mathematical Sciences and Technology, Norwegian University of Life Sciences, Ås, Norway; 2 Dept. of Psychology, University of Oslo, Oslo, Norway; 3 Dept. of Physics, University of Oslo, Oslo, Norway; The Research Center of Neurobiology-Neurophysiology of Marseille, France

## Abstract

Inhibitory interneurons (INs) in the lateral geniculate nucleus (LGN) provide both axonal and dendritic GABA output to thalamocortical relay cells (TCs). Distal parts of the IN dendrites often enter into complex arrangements known as triadic synapses, where the IN dendrite plays a dual role as postsynaptic to retinal input and presynaptic to TC dendrites. Dendritic GABA release can be triggered by retinal input, in a highly localized process that is functionally isolated from the soma, but can also be triggered by somatically elicited Ca^2+^-spikes and possibly by backpropagating action potentials. Ca^2+^-spikes in INs are predominantly mediated by T-type Ca^2+^-channels (T-channels). Due to the complex nature of the dendritic signalling, the function of the IN is likely to depend critically on how T-channels are distributed over the somatodendritic membrane (T-distribution). To study the relationship between the T-distribution and several IN response properties, we here run a series of simulations where we vary the T-distribution in a multicompartmental IN model with a realistic morphology. We find that the somatic response to somatic current injection is facilitated by a high T-channel density in the soma-region. Conversely, a high T-channel density in the distal dendritic region is found to facilitate dendritic signalling in *both* the outward direction (increases the response in distal dendrites to somatic input) and the inward direction (the soma responds stronger to distal synaptic input). The real T-distribution is likely to reflect a compromise between several neural functions, involving somatic response patterns and dendritic signalling.

## Introduction

A single neuron may contain a dozen or more different types of ion channels, including the traditional AP-generating Na^+^- and delayed-rectifier K^+^-channels and different types of Ca^2+^-channels. The way each type of ion channel is distributed over the somatodendritic membrane affects many aspects of neuronal function [1]–[5]. The functional advantage of a specific subcellular ion-channel distribution may depend on the morphology of the specific neuron, and on its role within the signalling network [1]. In the case of Ca^2+^-channels, the subcellular distribution may also impact on Ca^2+^-entry, which can trigger second messenger cascades that are important for a variety of cellular processes [6]–[9].

We focus in this paper on T-type Ca^2+^-channels (T-channels). T-channels typically activate when neurons are depolarized from relatively hyperpolarised resting potentials. Activation may lead to the generation of low-threshold Ca^2+^-spikes, which in turn can trigger bursts of action potentials (APs) [7], [10]. Also T-channels located in the dendrites can contribute to these bursts [11], [12]. In addition, dendritic T-channels are likely to amplify synaptic responses [13], [14], can provide a Ca^2+^ source for Ca^2+^-activated K^+^-channels [15], [16], and have been found in some cases to be involved in synaptic plasticity [17] and exocytosis [18], [19]. T-channels play an important role in a variety of cells [7], [10], [20]. Here, we study their role in neurons of the lateral geniculate nucleus (LGN) of the thalamus. Thalamocortical neurons (TCs) and local interneurons (INs) in the LGN both display T-channel mediated bursting [8], [15], [21]. Thalamic burst firing is thought to be involved in generating oscillatory brain activity during periods of sleep and generalized epilepsy [8], [22], [23]. In the LGN, burst firing has also been found to play a role during awake visual processing [24], [25].

Experimental studies have pointed in somewhat different directions regarding how T-channels are distributed on the dendrites of TCs [26]–[31]. The lack of consensus inspired a computational study which, rather than striving towards a direct prediction of the actual distribution of T-channels, explored the consequences that various T-channel distributions (T-distributions) would have for the response properties of TCs [32]. It was concluded that the propensity of the TC to elicit bursts of APs is highest if the T-channels predominantly are localized in the proximal dendrites. However, the study considered only somatic responses to somatic current injections, and it is uncertain to which degree the conclusions would hold for realistic, synaptic input [33]. Furthermore, it is conceivable that the subcellular distribution of ion channels may be important also for neural properties other than the somatic response generation.

The literature is also inconclusive regarding the T-distribution in INs. An experiment using Ca^2+^-imaging techniques suggested a density that increases linearly with distance (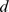) from the soma [26] in the proximal dendrites (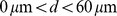). In this dendritic region, the amplitudes of [Ca^2+^]-transients due to T-currents were found to increase linearly with distance from soma. The findings were interpreted in favour of a predominantly dendritic localization of T-channels [8], [26]. A distal location of T-channels has found some indirect support in other experimental observations [8]. However, in a more recent experiment, based on genetic markers, no correlation was found between the local diameter of the dendrite and the expression of T-channels [29]. Given that the dendritic diameter tends to decrease with distance from soma, especially in the proximal region, these findings were taken to indicate that T-channels were uniformly distributed, i.e., have the same density over the whole somato-dendritic membrane [29]. To our knowledge, no study has yet explored the relationship between the T-distribution and the response properties of INs in a systematic manner.

INs have longer and thinner dendrites than TCs, and are less electronically compact [34], [35]. The functional consequence of the T-distribution can therefore be expected to be more critical in INs than in TCs. It is also likely to be more complex, as INs can provide GABA release both from axonal and dendritic terminals. Distal IN dendrites often form so called triadic synapses with axons of retinal ganglion cells and dendrites of TCs [36]–[39]. At these sites, the IN dendrite is both postsynaptic to retinal input and presynapic to TCs. To add to this complexity, GABA release from the IN terminals in these triads may be triggered in several ways: either directly by local synaptic input from the retina, in a process that is functionally decoupled from the IN soma [38], [40]–[42], or when the IN soma elicits Ca^2+^-spikes [43], and possibly also by backpropagating APs [43], [44]. Hence, the complex function of INs depends on the somatic firing properties as well as the two-way communication between the soma and distal dendritic sites, both of which are potentially influenced by the T-distribution.

In this work, we investigate how the distribution of T-channels (T-distribution) affects a set of functionally important response properties of INs, namely the somatic generation of regular APs (R1), the backpropagation of APs into distal dendrites (R2), the somatic generation of bursts of APs (R3), the spread of Ca^2+^-spikes into distal dendrites (R4), and the integration of synaptic input onto distal dendrites (R5). For all the response properties (R1-R5), our research question is essentially the same: assuming that we have a predefined amount of T-channels at our disposition, how should we distribute the T-channels over the somatodendritic membrane if the objective is to optimize for the respective properties? To answer this question, we use a previously developed multicompartment model of an LGN IN [45]. We run simulations using six adapted versions of this model, each having a different T-distribution, and compare their performance with respect to response properties (R1-R5).

The simulations, presented in the Results section, show that some of the response properties (R1 and R3) are facilitated by a T-distribution biased towards the proximal dendrites, whereas others (R2, R4, R5) are facilitated by a T-distribution biased towards the distal dendrites. In the Discussion we comment on these findings, and argue that the real T-distribution likely reflects a trade-off between several functional properties, rather than being optimized for a single function. Finally, the Methods section contains technical details concerning the computational model.

## Results

We explored the effect of the distribution of T-channels (T-distribution) on the following neural properties, **R1**: somatic, regular AP-firing **R2**: backpropagation of APs into distal dendrites, **R3**: somatic burst firing, **R4**: dendritic Ca^2+^-spikes, and **R5**: synaptic integration.

**Figure 1:**
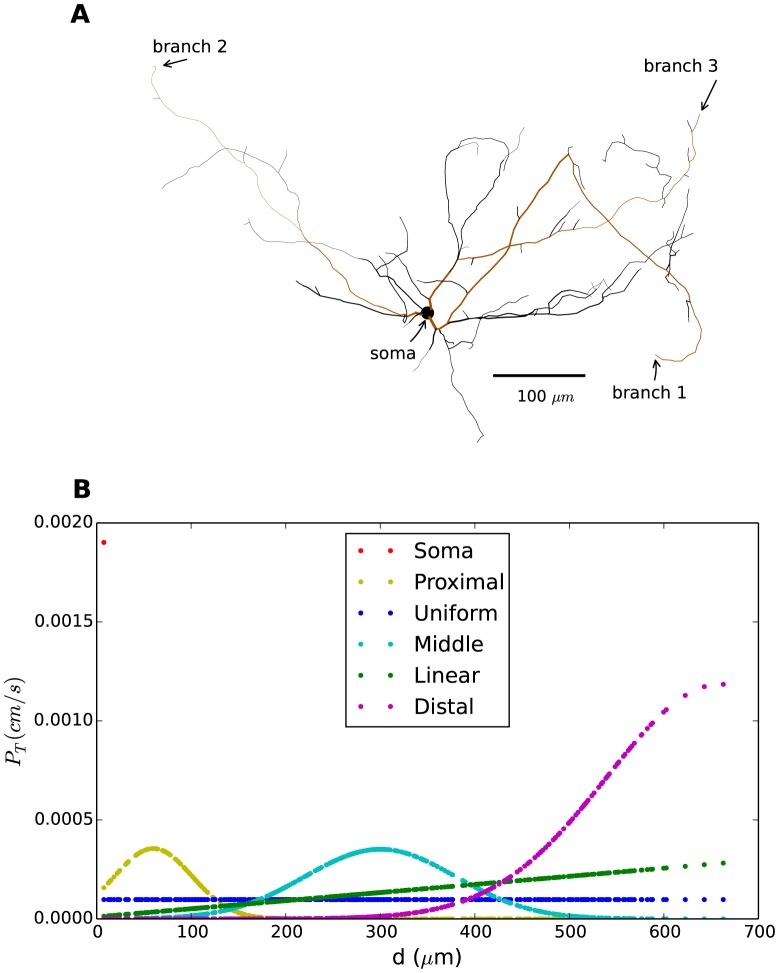
Morphology and T-channel distribution. (**A**) Morphology of IN based on 3D-reconstruction of a mouse interneuron. Arrows indicate the regions where the model receives input in the simulations: the soma and the most distal points in three independent branches (highlighted in brown). (**B**) Permeability (density) of T-channels (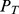) as a function of distance from the soma shown for six different T-distributions. Each dot indicates the value of 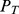 in a segment of the IN. The red dot (upper left corner) thus indicates the value of 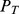 in the soma for the soma-distribution. 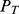 was normalized so that total permeability (summed over all dendrites) was the same in all distributions. Note that the areas under the curves are not identical. This is because there is a larger proportion of the total surface area at a proximal distance from the soma.

All simulations were performed on a multicompartment model of INs (adapted from [45]). The model was built using a realistic morphology based on a 3D-reconstruction of a mouse interneuron (Fig. 1A). It included standard Hodgkin-Huxley type Na^+^ and direct rectifying K^+^-channels for AP generation, in addition to the T-type Ca^2+^ channels. The model was presented in six versions, each characterized by a different T-distribution (Fig. 1B), namely (i) the *soma*-distribution, 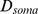 (red), where all T-channels were in the soma, (ii) the *proximal*-distribution, 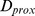 (yellow), where T-channels were clustered in the proximaldendritic region and in the soma, (iii) the *uniform*-distribution, 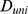 (blue), where T-channels were uniformly distributed over the membrane, (iv) the *middle*-distribution, 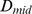 (cyan), where T-channels were clustered in dendritic regions halfway between the soma and the distal dendrites, (v) the *linear*-distribution, 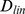 (green), where the density of T-channel increased linearly with distance from soma, and (vi) the *distal*-distribution, 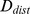 (magenta), where T-channels were clustered in distal dendrites. The T-channel distributions were normalized so that all model versions had the same total number of T-channels (see Methods section for further details).

Of the included distributions, 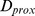, 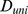 and 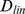 are the most likely candidates for representing the real distribution. 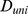 was in accordance with experimental data based on genetic markers [29], whereas both 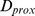 and 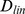 were in close agreement with experimental data based Ca^2+^-imaging [26]. In both 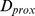 and 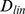, the T-density increased in a linear or close-to-linear fashion for the first 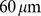 of the dendrites like in the experiments. With 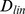, the initial linear trend was extrapolated to the full dendritic tree whereas 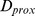, modelled with a Gaussian function, peaked at 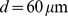 and decreased gradually with greater distances from the soma. Both experimental studies indicated a non-zero density of T-channels in both the soma and dendrites (at least in the proximal dendrites) of INs [26], [29]. In the current computational study, 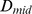 and 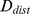 had a T-density close to zero in the soma, whereas 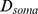 had a T-density of zero in the dendrites. These models are therefore unlikely to represent the real distributions, but were included in the study to explore the extreme limiting cases.

### Model properties

It has been suggested that IN firing under physiological conditions is predominantly controlled by synaptic input to the proximal dendrites [39]. In our simulations, IN responses (i.e., in the membrane potential, 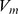) were instead evoked with somatic current injections, as this enabled us to compare our results with experimental literature using current-clamp recordings [15], [43], [45]. Experimentally, it has been found that activation of 1-4 synapses can evoke somatic excitatory post-synaptic current (EPSC) amplitudes between 30 and 600 pA in INs [43]. In all simulations presented in the following, the stimulus amplitude was within this physiologically realistic range. The IN model was able to qualitatively reproduce the two major types of response characteristics that have been observed in the IN, namely, 1) regular AP-firing, characterized by a steady stream of action potentials and 2) burst firing, characterized by bursts of APs that ride atop Ca^2+^-spikes [15], [23], [43], [45]–[47].

**Figure 2:**
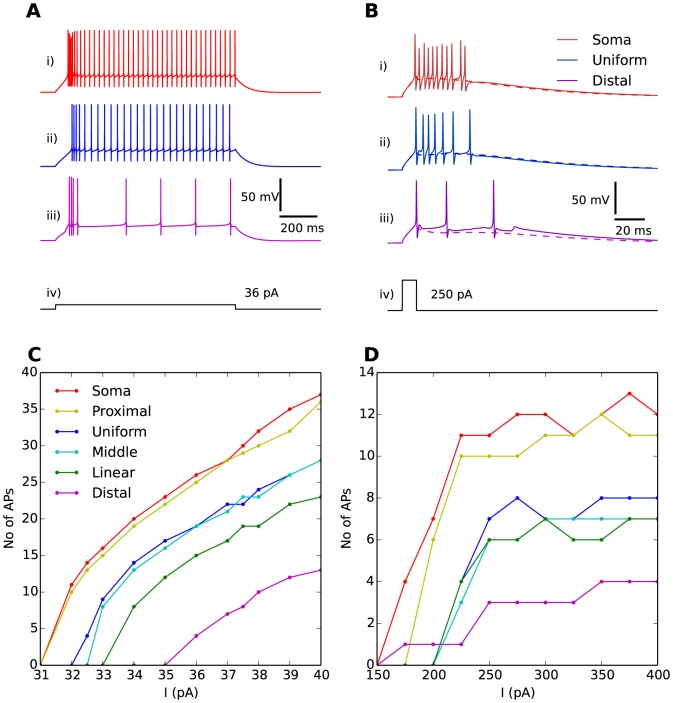
Response to somatic current injection. (**A**) A prolonged (1000 ms) stimulus protocol, 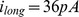 (A-iv), evoked an initial high AP-firing frequency phase, followed by a series of regularly spaced APs. (**B**) A brief (10 ms) stimulus protocol, 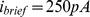 (B-iv), evoked a burst of APs. When AP firing was suppressed by setting the Na^+^-conductance to 0, the Ca^2+^-spike underlying the burst was revealed (dashed lines). The somatic response for three distributions are shown: the *soma* distribution (A-i, B-i), the *uniform* distribution (A-ii, B-ii), and the *distal* distribution (A-iii, B-iii). (**C**) Number of regular APs elicited during the last 700 ms of the stimulus period as a function of prolonged stimulus amplitude (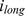) for all T-distributions. The regular AP-firing rate was facilitated by having T-channels in the proximity of the soma. (**D**) Number of APs elicited in bursts as a function of brief stimulus amplitude (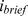) for all T-distributions. The bursting propensity was facilitated by having T-channels in the proximity of the soma.

**Figure 3:**
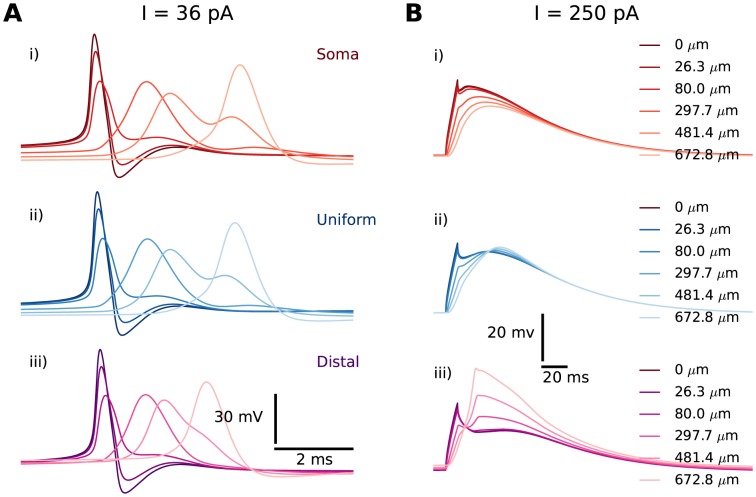
Regular APs and Ca^2+^-spikes invade distal dendrites. Propagation illustrated here in branch 1 for the *soma* (red), the *uniform* (blue) and the *distal* (magenta) distribution. (**A**) Backpropagating, regular APs shown at the soma (point of origin) and at selected locations along a single dendritic branch. Trains of regular APs were generated by a prolonged stimulus protocol (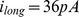 for 1000 ms) to the soma, and showed close-ups of the last AP in the train. (**B**) Ca^2+^-spikes shown at the soma and at selected locations along a single dendritic branch for selected distributions. Ca^2+^-spikes were evoked by a brief stimulus protocol (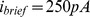 for 10 ms), and with Na^+^-conductances set to 0 to suppress AP firing. Curves were graded from dark colours (close to the soma) to light colours (far from the soma).

A prolonged current injection with a relatively low amplitude (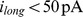) evoked an initial phase of rapid AP-firing, followed by slower, regular AP-firing (Fig. 2A). The response to the prolonged stimulus resembled that observed experimentally [45]–[47]. Single APs successfully backpropagated into the distal dendrites of the IN (Fig. 3A), as has also been seen in experiments [43], [44]. A brief current injection with high amplitude (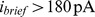) evoked a pronounced burst of APs (Fig. 2B). The response to the brief stimulus resembled that observed experimentally in [43]. The burst rode atop a Ca^2+^-spike mediated by T-channels [15], [23], [45]. The Ca^2+^-spike could be seen in isolation when AP-firing was suppressed by setting the Na^+^ conductances to zero (Fig. 2B, dashed lines). The Ca^2+^-spikes successfully invaded the dendritic arbor of the INs (Fig. 3B), as in [43].

A broad range of stimulus amplitudes and different T-distributions produced qualitatively similar responses to those shown in the representative examples in Figs.2 and 3. These response patterns and their dependence on the T-distribution are further analyzed below.

### Regular APs

Regular AP-firing (Fig. 2A) was evoked using a prolonged stimulus protocol, i.e., a 1000 ms current injection (Fig. 2A-iv). The initial, irregular high-AP-frequency phase of the response typically lasted between 100 and 200 ms, and was due to the activation of T-channels. This initial high frequency phase was ignored in the subsequent study where we focus on the regular AP-firing frequency (R1) and the dendritic backpropagation of a single, regular AP (R2). The same current injection 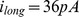 was used in all simulations of AP-propagation to evoke regular AP-firing.

#### T-distribution affects regular AP firing

For a simple, quantitative measure of the effect of T-distribution on somatic propensity to fire regular (non-burst) APs (R1), we counted the number of regular APs elicited during the last 700 ms of the (1000 ms) stimulus period (we started the count 300 ms after the stimulus onset to make sure that the neuron had settled into regular AP-firing). For example, a stimulus 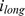 of amplitude 36 pA gave a count of 4 regular APs in that time range with 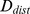 (Fig. 2A-iii).

When the experiment was repeated for different stimulus amplitudes and T-distributions, we obtained the input/output (I/O)-curves in Fig. 2C. For all T-distributions, the regular AP-firing rate increased with stimulus amplitude, with slopes of around 4 APs/pA (this corresponds to about 5-6 Hz/pA, as we counted in a 700 ms period). In a previous modelling study, we showed INs are likely to possess modulatory mechanisms that would reduce the AP firing rate [45]. As no modulatory mechanisms were included in our current model, it generally showed a higher AP-firing frequency than that observed experimentally in INs, where I/O-curves typically have slopes that vary from about 0.2-0.5 Hz/pA [45], [47] and up to about 3Hz/pA [46]. We do not believe, however, that the high AP-firing rates obtained with our model would influence our results regarding the role of T-channels.

Regardless of the value used for stimulus amplitude 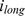, the highest firing rate was consistently achieved with 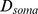 and 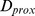, while 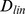 and 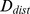 gave the lowest firing rate. This indicates that regular somatic firing of regular APs is facilitated by having T-channels in close proximity to the soma.

#### Successful AP-backpropagation does not depend on T-distribution

In the following simulations, the same current injection 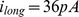 was used in all simulations of AP-propagation to evoke regular AP-firing. We studied the effect of the T-distribution on the backpropagation of a single, regular AP (R2). In order to circumvent any distortion effect that might be related to the initial Ca^2+^-spike, we focused on the last AP in the series of regular APs (obtained by using the prolonged stimulus protocol) and observed its propagation along different dendritic branches.

Fig. 3A shows, in selected segments along a single branch, how the amplitude and shape of the (last) single AP evolved as it (back-) propagated from the soma towards the distal dendrites. The somatic AP-shapes obtained with 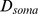, 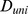 and 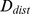 (Fig. 3A(i–iii)) were as good as identical. AP-backpropagation was also very similar in the different distributions. In all cases, the AP experienced a broadening and a decrease in amplitude upon its propagation away from the soma.

**Figure 4:**
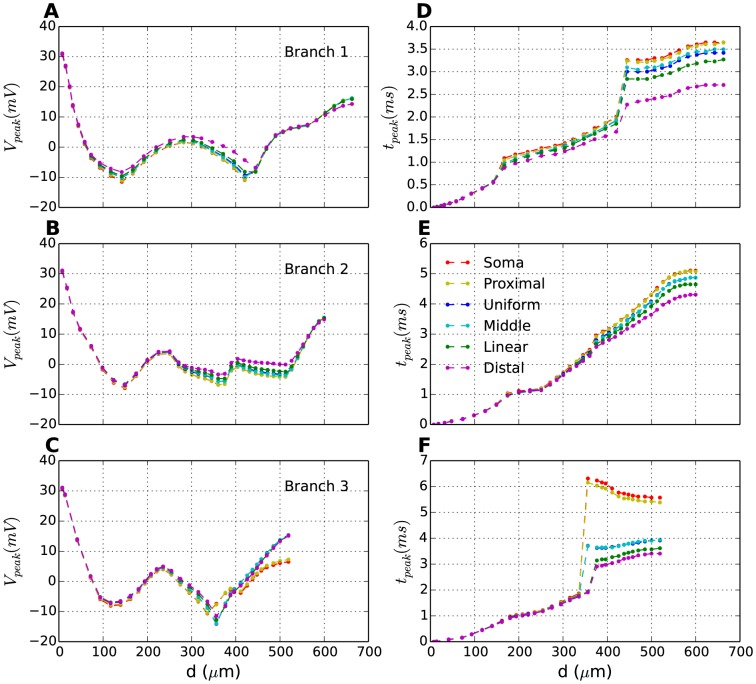
Effect of T-distribution on AP backpropagation. (**A**–**C**) In each segment of a selected branch, the peak amplitude of last action potential (Fig. 2A) (left panel) and (**D**–**F**) the time taken from soma to reach peak amplitude (right panel) are plotted as a function of distance from soma (peak time, 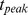, was plotted relative to the time of the somatic AP-peak (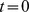)). While the distribution does not have a significant effect on peak amplitude of last potential (A–C), it does affect the speed of propagation as shown in D–F. The signal travels faster in distributions where the density of 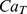 channels increases in distal dendrites.

Fig. 4 summarizes AP-backpropagation in three different dendritic branches, and for all T-distributions. Fig. 4(A–C) shows the AP-amplitude (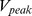) as a function of distance from the soma in three different branches (Fig. 1A). The panels (A–C) in Fig. 4 represent the different branches, while the six curves in each panel represent different T-distributions. The exact evolution of the AP amplitude will depend on complex morphological features of the branching dendrites. We do not explore this in further detail here, but focus solely on how AP-propagation is influenced by T-distribution.

The exact shape of the attenuation profile depends on branch-specific morphological features of the interneuron (see Fig. S3 for a detailed explanation). Despite morphological variation between the branches (compare panels A–C), AP propagation followed the same general trend. In the proximal dendrites, the AP amplitude decreased monotonically with distance from soma, from about 26.7 mV in the soma to between −10 mV and −15 mV at a distance of around 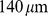 from the soma. At intermediate distances 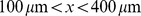, 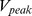 varied between −15 and 0 mV. In the distal dendrites 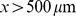, the AP amplitude increased with distance from soma, reaching an amplitude between 8 and 16 mV at the end point.

A comparison between 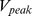-curves for each individual branch (A–C) shows that the T-distribution had a relatively small impact on 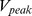. Also, there was no trend in how the T-distributions influenced the local 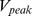. For example, 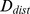 gave the lowest 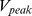 at the endpoint of one branch (Fig. 4A), and the highest 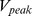 at the endpoint of another branch (Fig. 4C). We conclude that the T-distribution had no clear effect on the shape and amplitude of backpropagating APs.

#### Dendritic T-channels increase AP propagation speed

Interestingly, we found that the T-distribution did affect the propagation speed of APs (R2). The time at which AP-peak value is reached (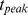) in a given segment is plotted as a function of distance from the soma in branches 1–3 (Fig. 4(D–F)). In all branches, backpropagating APs took consistently less time to reach dendritic endpoints when the T-density was high in those regions (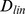 and 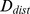). In general, backpropagation took longer when the T-density was low in the distal regions (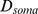 and 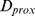). In one case, however, where another set of initial parameters was used (see Fig. S5), 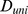 was observed to be slower than all the other distributions in branch 3.

### Bursts and Ca^2+^-spikes

In this section, we study burst firing, evoked here with a brief (10 ms) current injection in the soma (Fig. 2B-iv). During the bursts, the AP amplitude varied, a phenomenon observed both experimentally and in previous modelling studies [15], [45]. We here focus on the effect of the T-distribution on the burst (R3), the magnitude of the Ca^2+^-spike, and the way that the Ca^2+^-spike is conveyed to distal dendrites (R4). For a quantitative measure of the effect of the T-distribution on the burst for a given stimulus, we use the number of APs riding the crest of the low-threshold Ca^2+^-spike (Fig. 2B).

#### Effect of T-distribution and stimulus amplitude on somatic burst firing

Fig. 2D shows the number of APs elicited during a burst (R3) as a function of the stimulus amplitude, for all the different T-distributions. For strong current injections (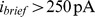), the number of APs in the burst tended to saturate beyond a certain stimulus amplitude. This suggests that the T-distribution sets an upper limit to the the number of APs that can be elicited by somatic bursts, and thus elucidates the fundamental role of T-channels in burst generation. The effect of the T-distribution on the bursts was similar to that observed for regular AP-firing. Above a certain stimulus amplitude (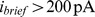), we consistently observed that the model versions with T-channels close to the soma (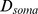 and 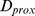) elicited the bursts with the highest number of APs. Qualitatively similar results have been found previously, in a computational study of TCs [32] where the somatic bursting propensity was also found to be facilitated by a high T-density in the proximal dendritic region.

We conclude that the propensity of the soma to elicit bursts of APs (as well as regular APs) is facilitated by a T-distribution biased towards the soma-region. These findings were not unexpected. As the stimulus injection was applied to the soma, it seems reasonable that a high density of T-channels in the proximity of (or in) this compartment would boost the local response.

#### Effect of T-distribution on somatic Ca^2+^-spikes

It is known that Ca^2+^-spikes can trigger dendritic GABA-release in INs, even when AP-firing is suppressed by the Na^+^-channel blocker TTX [43]. GABA-release triggered by Ca^2+^-spikes has been found to have a longer duration compared to the (axonal and possibly dendritic) GABA-release triggered by single APs [43]. This implies that the Ca^2+^-spike has an important functional role beyond that of mediating bursts of APs, and motivated us to conduct a further study of the Ca^2+^-spike in isolation.

**Figure 5:**
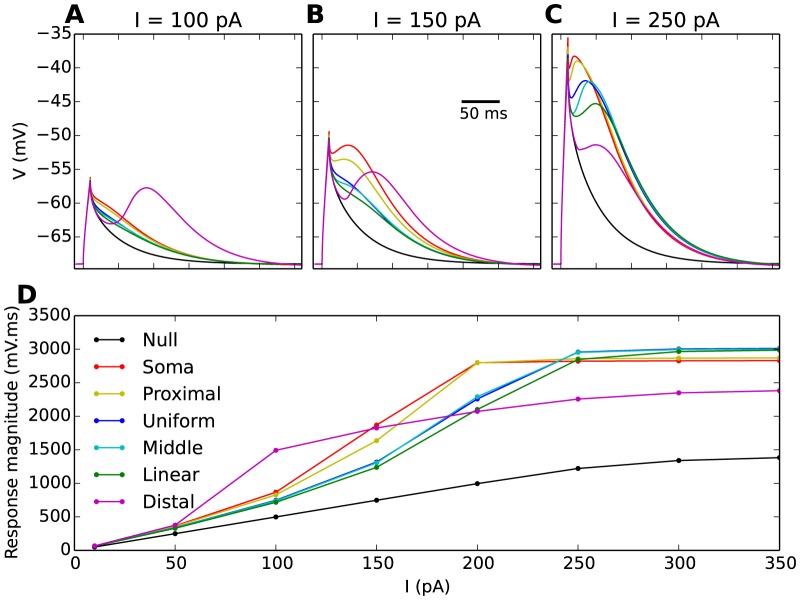
Ca^2+^-spikes in the soma for all T-distributions. (**A**–**C**) Ca^2+^-spikes, evoked by 10 ms somatic current injections are illustrated here for stimulus amplitudes, 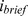, of 100, 150 and 250 pA. AP firing was suppressed by setting the Na^+^-conductance to 0. The Ca^2+^-spikes varied significantly between the different T-distributions (differently coloured curves, as indicated by legend in **D**). (**D**) Magnitude of Ca^2+^-spikes as a function of the stimulus amplitude for all T-distributions. For technical reasons (a Ca^2+^-spike peak amplitude could not be defined for all cases), the response magnitude refers to the area under the response curve. For reference, a response curve for a null distribution (black curve), a model devoid of T-channels, was included in all panels.

In the following simulations (Fig. 5), the Na^+^ conductance was therefore set to zero so as to suppress the APs in the burst. Brief current injections then evoked Ca^2+^-spikes. In order to gauge the effect of the T-channels on the response, we compared the somatic voltage response of the different model distributions with the response obtained when no T-channels were included in the model (referred to as the null-distribution, black line).

Simulations were run for different stimulus amplitudes. In all cases (Fig. 5A–C), the brief stimulus caused an initial peak in 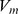 after 10 ms. After stimulus offset, 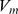 initially started to decay towards the resting potential (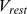). However, the relatively slow activation of the T-channels gave rise to an inward T-current which interfered with the passive decay. To describe the observed responses, we make a distinction between *full* and *partial* Ca^2+^-spikes. When the T-current proceeding from the stimulus was strong enough to evoke a second, post-stimulus depolarization, and thus a second local maxima in 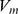 at some point after the stimulus offset (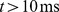), we considered that we had a full Ca^2+^-spike. Full Ca^2+^-spikes were, in all T-distribution models, evoked only for strong stimulus amplitudes (Fig. 5C). For weaker stimulus amplitudes (Fig. 5A–B), only a subset of the distributions elicited full Ca^2+^-spikes. For example, with 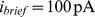, only 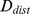 elicited a full Ca^2+^-spike (Fig. 5A). For the other distributions, the T-current merely slowed down the decay of 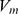 towards the resting potential, compared to what we would expect from a purely passive response. We refer to this kind of T-channel mediated response as partial Ca^2+^-spikes.

For a quantitative measure of the magnitude of the Ca^2+^-spike, we chose to use the area under the response curve. This was partly practically motivated as, e.g., a quantification in terms of the peak amplitude could not be defined in the case of partial Ca^2+^-spikes. However, as we know that Ca^2+^-spikes trigger dendritic actions distinct from that of single APs [43], these actions likely depend on the time-course of the depolarization, and not only the peak amplitude. As the area under the response curve depends on both these aspects, we reasoned that it represented a functionally relevant quantification of the magnitude of the Ca^2+^-spike.

In all model versions, the Ca^2+^-spike area increased with increasing stimulus injection, but approached saturation and increased little when 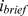 was increased beyond 300 pA (Fig. 5D). Interestingly, the T-distribution that gave the biggest Ca^2+^-spike response, depended on the stimulus injection. For a relatively weak stimulus (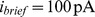), 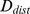 gave the biggest response area. For stimuli at intermediate strength (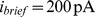), 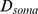 and 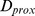 gave the biggest response area. Finally, for strong stimuli (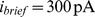), 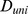, 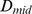 and 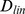 gave the biggest response area. We do not go further into the details of this, but we make use of this analysis below, when we study how the Ca^2+^-spike is conveyed from the soma to distal dendrites.

#### T-distribution affects the way somatic Ca^2+^-spikes are conveyed to distal dendrites

Next, we investigated the influence of the T-distribution on how well somatically elicited Ca^2+^-spikes were conveyed to distal dendritic sites (R4). In the case of AP backpropagation, it was clear that the AP originated in the soma, and then propagated towards the distal dendrites (Figs. 3 and 4). The time-aspect of dendritic Ca^2+^-spikes was more complex. As T-channels have much slower kinetics than Na^+^-channels, T-channels over the entire dendritic tree had overlapping activation time, and contributed simultaneously to the generation of Ca^2+^-spikes. In the case of Ca^2+^-spikes, it would therefore not be appropriate to speak of *propagation*. For instance, for 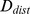, the Ca^2+^-spike reached its peak value earlier in the distal dendrite than in the soma, although the current injection was applied to the soma (Fig. 3D). We did not explore the time-aspect of Ca^2+^-spikes further, but from now on focus on their magnitude.

**Figure 6:**
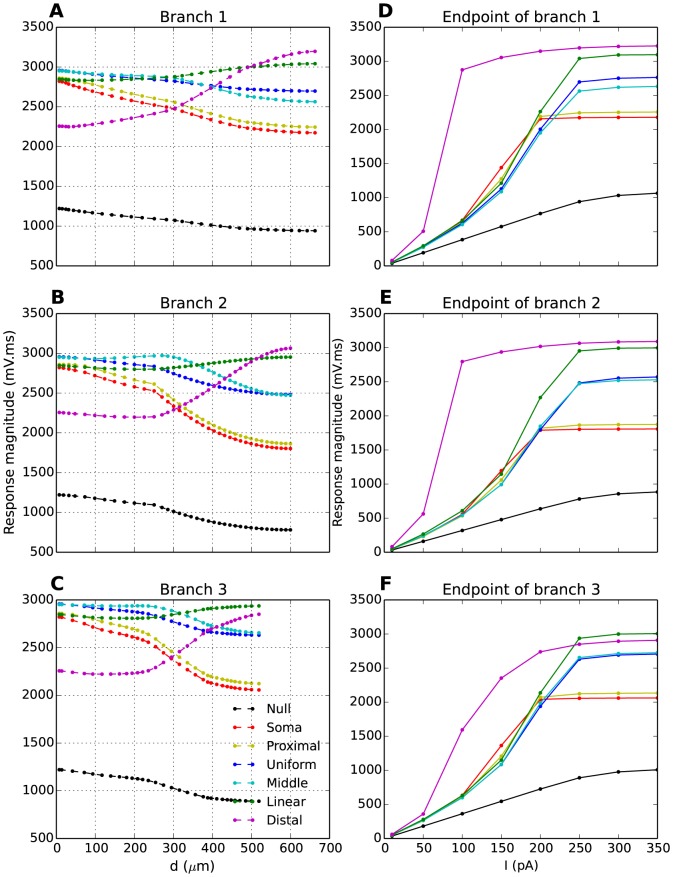
Ca^2+^-spikes in the dendritic tree. (**A-C**) Magnitude of Ca^2+^-spikes as a function of distance to the soma. The Ca^2+^-spike magnitude clearly reflected the underlying T-distribution (different coloured curves, as indicated by legend). Ca^2+^-spikes were evoked by brief (10 ms) current injections with stimulus amplitude (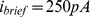). (**D-F**) Magnitude of Ca^2+^-spikes at dendritic endpoints as a function of stimulus amplitude 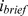. The magnitude was always biggest for T-distributions with a high distal T-density. (A-F) In all cases, AP firing was suppressed by setting the Na^+^-conductance to 0. Response magnitude was defined as the area under the response curve. The different panels in the vertical direction represent three different dendritic branches.

In Fig. 6A–C, we have plotted the Ca^2+^-spike magnitude as function of the distance from soma in three different branches. The local magnitude of the Ca^2+^-spikes depended on two factors: (i) the proximity to the injection site, and (ii) the local T-density. The first factor (i) explains the results obtained with 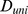 (blue line). In that case, the Ca^2+^-spike-magnitude decreased with distance from soma, almost in parallel to how the passive response obtained with the null-distribution decreased with distance (black line). In case of 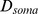 and 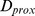 (red and yellow lines), the Ca^2+^-spike-magnitude decreased more steeply with distance from soma, due to the additional effect (ii) of decreasing T-density. In case of 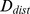 or 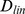 (purple and green lines), the second factor (ii) dominated over the first (i), and the Ca^2+^-spike response magnitude increased with distance from the soma, giving a maximum value at dendritic endpoints, where the T-density was highest. Finally, in case of 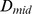 (cyan line), the Ca^2+^-spike response magnitude was either highest in the soma (Fig. 6(A,C)), where the stimulus was applied, or around 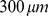 from the soma, where the T-density was highest (Fig. 6B). For distances greater than 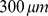 from the soma, the Ca^2+^-spike-magnitude decreased slowly with distance from soma.

Next, we explored how the trends identified in Fig. 6A–C depended on the amplitude of the somatic current injection. As we were mainly interested in how Ca^2+^-spikes were conveyed to dendritic endpoints, i.e., the typical locations of triadic synapses, we limited the analysis to explore the Ca^2+^-spike-magnitude at the tips of the three dendritic branches (Fig. 6D–F). The trends that we identified in Fig. 6 A–C were robust for all stimulus injection above a certain threshold (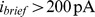). Below this threshold, the Ca^2+^-spike-magnitudes were quite similar in all model versions, except in 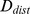, where the Ca^2+^-spike had a high amplitude in the distal dendrites, even for very weak stimuli (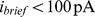). However, we recall from Fig. 5 that, for 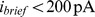, not all model versions elicited full Ca^2+^-spikes with well defined peaks in 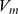. We therefore regard the results obtained with stimuli above 200 pA as most relevant for our study of Ca^2+^-spikes.

We conclude that, for brief somatic current injections strong enough to evoke full Ca^2+^-spikes in all model versions, the Ca^2+^-spike-magnitude in the tips of the dendrites was facilitated by having a high distal density of T-channels (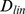 and 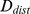). Similar results were found when we used another parametrization (P1) of the model (Figs. S5–S7).

### Synaptic integration

Finally, we investigated the impact of the T-distribution on synaptic integration (R5). Excitatory postsynaptic potentials (EPSPs) were evoked by a AMPA synapse inserted at the end points in one of the three branches (1, 2 and 3 in Fig. 1A). The synapse was adapted to experimental data for AMPA-synapses on thalamocortical relay neurons [41], and is described in further detail in the Methods section. To isolate effects due to T-channels, the sodium conductance was blocked. However, qualitatively similar results were obtained when the sodium conductance was not blocked (Figs. S1 and S2).

#### Impact of the T-distribution on synaptic integration

**Figure 7:**
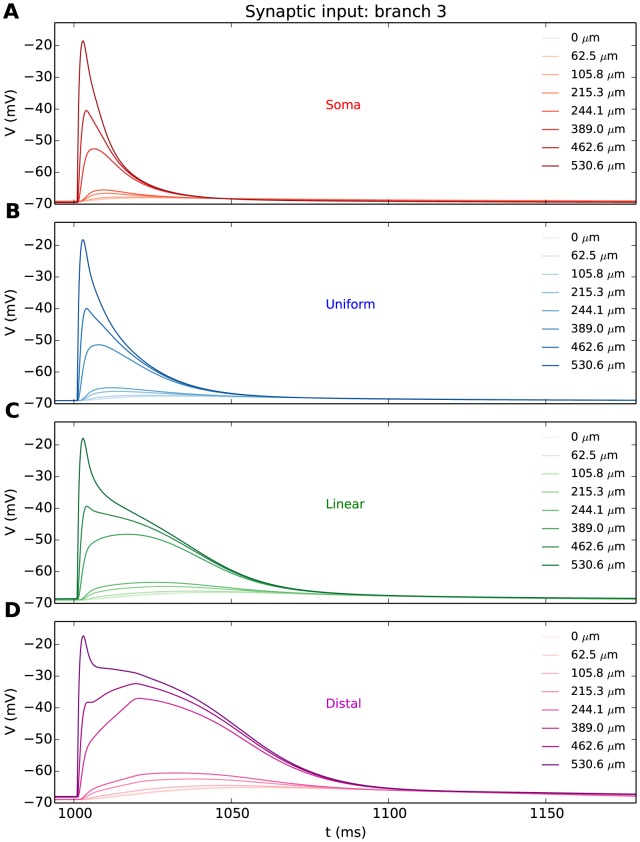
EPSPs obtained with different T-distributions. (**A-D**) EPSPs at different locations along a single dendritic branch (branch 3), as a response to synaptic input (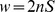) applied to the dendritic endpoint. Different panels represent different T-distributions, as indicated. EPSPs were attenuated upon propagation from the synapse (dark coloured curves) to the soma (light coloured curves). The Na^+^-conductance was set to 0 to suppress the dendritic Na^+^-spikes that would have been evoked in distributions with a high density of T-channels close to the synapse. The figure legends indicate distance from soma.

Synaptic activation evoked a local EPSP at the synaptic site, which eventually resulted in a voltage deflection in the soma. The evolution of the EPSP is illustrated for selected segments along branch 3 (Fig. 7) for the soma-, uniform-, linear-, and distal T-distributions. The T-distribution had a clear effect on the local, dendritic response. When all the T-channels were in the soma, the local dendritic EPSP to the synaptic input had a peak amplitude of about -20 mV (Fig. 7A). A non-zero T-density in the distal dendrite significantly prolonged the duration of the local EPSP-response at the synaptic site (Fig. 7B–D). A similar role for dendritic T-channels has been proposed in an earlier, general computational study [48].

**Figure 8:**
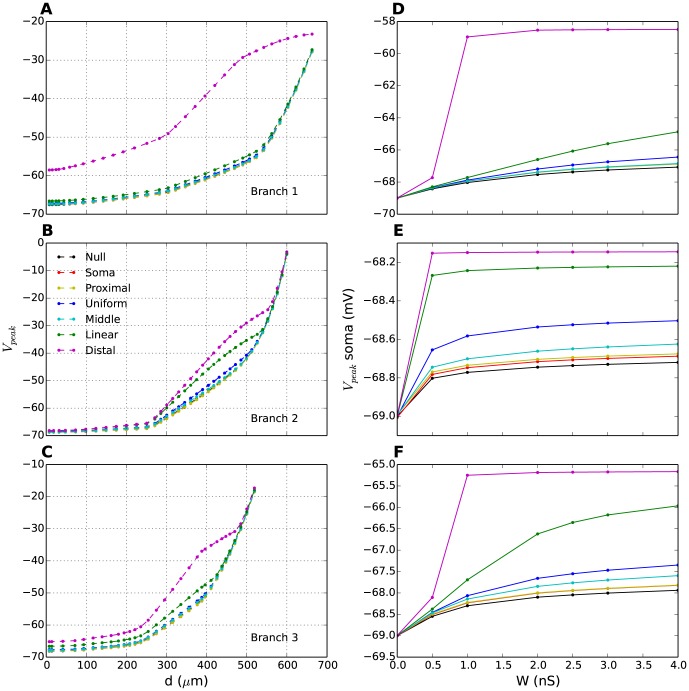
EPSP amplitudes for different T-distributions. (**A-C**) EPSP amplitude (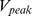) as a function of distance from soma in 3 different branches (each panel represents a distinct branch). In each branch, synaptic input was applied to the dendritic endpoint. For all distributions, EPSP amplitudes decreased upon propagation towards the soma. (**D-F**) Somatic EPSP amplitude as a function of synaptic weight, 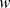. The somatic EPSPs had bigger amplitude for T-distributions with a high density of T-channels close to the synapse. The synaptic weight in (A-C) was 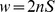. (A-F) Different coloured lines correspond to different T-distributions. Black lines (null distribution) correspond to the case without T-channels.

The propagation of the EPSP towards the soma is summarized for all six distributions in Figs. 8A–C, which show how the peak amplitude of the signal vary with distance from the soma in 3 different branches. While the duration of the EPSPs (Fig. 7) varied with different T-distributions, their amplitudes at the synaptic sites were similar. The attenuation profiles of the signals as they propagated towards the soma were similar in appearance except for 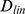 and 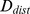. In the case of 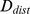 especially, there was a significant boost in the response partly caused by the presence of T-channels along the way. We note here that the signal boost is far more significant in branches 1 and 3 than in branch 2, showing that it is affected not only by the distribution of the T-channels, but also by morphological features. Branch 2 has fewer ramifications (i.e. smaller membrane area) and thus fewer T-channels in the distal regions than branches 1 and 3 where the signal is given an additional boost as a result of backpropagation from nearby dendritic branches. This explains the smaller difference between the linear and the distal distribution for this particular branch. In fact, when we used another parametrization P1 (see Methods), 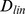 turned out to be more efficient than 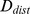 for this branch (see Fig. S9).

Due to the low-pass filtering properties of the dendrites, the most enduring EPSPs experienced the least attenuation during propagation. This is apparent in Fig. 8D–F, which shows EPSP-amplitude in the soma as a function of the synaptic strength. The general trend was that the EPSP amplitude in the soma was higher when the T-density was high in the distal dendrites. This trend was independent of the synaptic strength.

It hence turned out to be more efficient to position the T-channels locally at the synaptic site, thus boosting the EPSP *before* integration than to have a high T-density in the soma thereby boosting the incoming EPSP response *when* it arrived.

## Discussion

We have explored how the subcellular distribution of T-channels influences five different response properties (R1-R5) of a multicompartmental model of LGN INs. Our research question was essentially: given a limited number of T-channels, how should they be distributed over the somatodendritic membrane if the purpose of the distribution were to enhance the respective properties?

Our study showed that different T-distributions were optimal for different properties. The somatic response to somatic current injections was facilitated by a high T-channel density in the soma or proximal dendritic region. A high T-density near the soma thus gave rise to a higher regular AP-firing rate (R1) and a higher number of APs in a burst (R3). On the other hand, a high T-density in the distal dendrites facilitated the conveyance of somatic signals to distal dendrites, by increasing AP propagation speed (R2) and increasing the voltage deflections evoked in distal dendrites by somatic Ca^2+^-spikes. Interestingly, a high distal T-density also facilitated the somatic integration of distal synaptic input(R5). Thus, there was an asymmetry in the signalling between the soma and distal dendrites: For signals propagating outwards, the response in a distal dendrite was maximized by having a high local T-density boost the signal *when* it arrived its distal destination. For dendritic input, the response in the soma was maximized by having a high local T-density boost the signal *before* it propagated towards its destination. Hence, the somatic response to somatic current injections was facilitated by a high T-density in the soma-region, whereas communication in *both* directions between the soma and distal dendrites was facilitated by a high T-density in the distal dendrites.

Below we revisit the response properties (R1-R5) and comment on their putative importance for the function of INs, and the degree to which we may expect the T-distribution to be a key factor in controlling them. We also discuss the possible impact of some of the ion channels that were not included in our model [15], [45], [47], [49] on these properties.

### R1: Somatic, regular AP-firing

It seems unlikely that the T-distribution should be adapted to yield a certain regular AP-firing rate in INs. In our simulations, the increase in firing rate gained from changing between the two extreme T-distributions (from 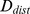 to 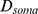) could also be achieved with a relatively small (20%) increase in the stimulus amplitude, suggesting that the AP-firing rate could be controlled by any depolarizing current. Based on general knowledge of neural firing properties, we would expect it to chiefly be controlled by Na^+^- and K^+^-channels [50]. Ca^2+^-dependent afterhyperpolarization currents may also be involved in reducing the IN firing rate [45].

Several other membrane mechanisms (not included in the current model) may also affect the AP-firing rate. For example, L-type Ca^2+^ channels (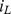) have been shown to activate through depolarizations positive to -35mV [26], [49]. This suggests that they should predominantly open during AP firing. Ca^2+^ influx through 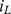 could then provide a source for activation of Ca^2+^-dependent afterhyperpolarization channels (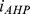), which could reduce the AP-firing frequency in thalamic neurons [45], [51].

### R2: Backpropagation of APs into distal dendrites

We showed that AP-backpropagation could be speeded up with a high T-density in distal dendrites (Fig. 4). To our knowledge, this is the first study that has predicted an impact of T-channels on AP-propagation speed. It is however unlikely that its potential impact on the AP-propagation speed is the key role of the T-distribution. Generally, the most important prerequisite for AP-backpropagation is the dendritic Na^+^-conductance [44], [52]. Other conductances could play a role. For example, INs possess A-type K^+^-channels (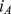) [49]. This ion channel type has been found to influence AP-backpropagation in hippocampal neurons [53]. It also remains unclear whether backpropagating APs play a role in mediating IN output [43].

### R3: Somatic burst firing

It has been established that T-channels are the main mediators of bursts in INs [8], [15], [21], [45], [49], although contributions from other ion channels have been found. Our simulations showed that, beyond a certain stimulus, the number of APs elicited during a burst tended to saturate towards a maximum value, which depended on the T-distribution (Fig. 2). This highlights the unique role of T-channels in burst generation. The T-distribution is thus likely to have adapted, at least partly, to regulate the somatic bursting pattern, as has been suggested for other thalamic neurons [11], [32].

As in TCs [32], a high T-density in the proximal dendrites resulted in a greater propensity for somatic bursting. However, in INs, the T-distribution may not be optimized solely to enhance the number of APs in a burst. Experimentally recorded IN bursts typically having a lower intraburst AP-frequency and a longer duration than TC bursts [15], [21], [46], [47], are closer in resemblance to thalamic reticular (RE) neurons. In RE neurons, which have been likened to INs [54], prolonged bursts have been explained by a high T-density in distal dendrites [11]. Our simulations also indicate that a reduction in the intraburst AP-firing frequency and a prolonged burst-duration may result from moving T-channels away from the proximal region towards the distal dendrites (Fig. 2). On the other hand, differences in burst shapes between INs and TCs may also be explained by differences in total T-conductance and T-channel kinetics [21]. In fact, characteristics of IN bursts and the numbers of APs therein tend to vary quite significantly between different INs [15], [49]. It is therefore difficult to use a quantitative comparison between the simulated bursts in Fig. 2 and experimentally observed bursts to deduce the most likely T-distribution.

Somatic burst firing may also depend on ion channels not included in this study. Ca^2+^-activated non-selective cation channels (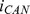), for example, have been found to prolong the duration of bursts in INs, and in some cases evoke plateau-potentials [15]. As 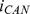 in INs is thought to be activated predominantly by Ca^2+^ entering through T-channels [15], its presence could serve to amplify T-channel mediated effects, and make the functional consequences of the T-distribution even more pronounced. A hyperpolarization-activated non-specific cation channel (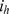) has been identified in INs [45], [55]. 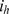 has been found to contribute to the generation of rebound bursts [45], i.e., bursts that the IN elicit when it is released from holding potentials far more hyperpolarized than the resting potential. However, due to the strong hyperpolarizations required to activate 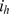 [45], it is unlikely that 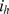 would be active in the situations that we studied in the current work.

Although most studies have indicated that burst firing in INs is predominantly mediated by T-channels [8], [15], [21], [23], [26], [46], [49], one experiment has observed bursts that could be abolished by the appliance of the L-type Ca^2+^ channels blocker nimodipine [43], suggesting that these Ca^2+^-spikes and bursts were mediated by 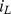. Nimodipine has also been found to have a strong impact on dendritic GABA-release [43], [56], and post-synaptic NMDA-responses [57] in INs suggesting that 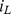 may have several functional roles in INs that may overlap with those of T-channels. The bursts observed in [43] were evoked by brief (10 ms) stimulus injections to the soma, and showed a striking similarity (in terms of amplitude, duration and number of APs) to the bursts that we obtained in our simulations when we used the same stimulus protocol (compare Fig. 2 with Fig. 1 in [43]). In our study, bursts were a robust response feature, generated by T-channels that were modelled with activation kinetics based on experimental data from INs [21], [45]. This raises questions regarding the distinct roles of T-channels and 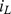 for IN signalling. Bursts mediated by 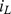 also seem to somehow challenge the high depolarization required for 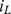 activation in previous experiments [26], [49]. A potential resolution to these apparently conflicting findings could be that nimodipine also interacts with T-channels, and that some of the effects of nimodipine appliance can be ascribed to the suppression of T-channel mediated activity. Experimental evidence that nimodipine may act on T-channels is sparse, but a nimodipine-induced reduction of T-channel activity has been demonstrated in neurons of the lateral dorsal nucleus of the thalamus [58]. We do not know if this is a plausible hypothesis in INs, but rather wish to pose it as an open question to the research community.

### R4: Dendritic spread of Ca^2+^-spikes

The Ca^2+^-spikes underlying the bursts, and the manner in which these are conveyed to distant sites in IN dendrites is of particular interest. Dendritic Ca^2+^-spikes have been known to trigger GABA-release from IN dendrites, even when AP-firing was suppressed by the Na^+^-channel blocker TTX [43]. Furthermore, GABA-release has been found to have a longer duration when triggered by Ca^2+^-spikes than when triggered by single APs [43]. The T-distribution could therefore have adapted in a way that guarantees that Ca^2+^-spikes are well conveyed to the distal dendrites. The T-distributions that were found to favour this function did not favour somatic bursting (R3) and vice-versa. On one hand, a high proximal T-density increases the number of APs in the burst (Fig. 2), and thus the axonal IN output. On the other hand, a high distal T-density increases the response magnitude of Ca^2+^-spikes in distal dendrites (Fig. 6), and putatively the dendritic output of INs. More detailed insight into the action of dendritic Ca^2+^-spikes will have to await experimental identification of the detailed mechanisms behind dendritic GABA-release, which are currently unknown. It has been suggested that GABA-release is controlled by a local voltage threshold [59]. A second possibility, that GABA-release rather depend on widespread (i.e., not highly localized to presynaptic terminals), intracellular Ca^2+^-dynamics, has also been discussed [43], [60]. Ca^2+^ entering through T-channels has been shown to be involved in exocytosis in some neurons [18], [19], yet it is unknown whether this is the case in thalamic neurons. Another possible action of dendritic Ca^2+^-spikes could be to provide depolarizations necessary for the relief of the voltage-dependent Mg^2+^-block of NMDA-receptors, which could induce changes in the post-synaptic strength [61], [62].

Note that we have focused primarily on signalling between the soma and most distal dendritic endpoints. However, it is possible that Ca^2+^-spike triggered GABA-release may occur throughout the dendritic tree. If we had looked at Ca^2+^-spikes from more proximal dendritic sites, our conclusions regarding the optimal T-distribution would have been different.

### R5: Synaptic integration

The response to distal synaptic input is a complex topic. A high distal T-density was shown to boost and prolong the EPSPs in the soma (Fig. 8), thus enhancing the integration of distal synaptic input (Fig. 7). This has been proposed earlier as a key functional role of distal T-channels in RE neurons [63]. However, because the dendrites in INs are long and leaky, somatic activity of INs could be controlled more efficiently by synapses positioned at more proximal locations [39].

Also, triadic synapses in distal dendrites may release GABA as a response to local, synaptic input from the retina, i.e., without requiring involvement from the soma of INs [36], [39], [41], [43], [57], [59]. In fact, it might even be undesirable to have strong somatic EPSPs evoked by dendritic input to the triads, as the purpose of their distal location may be to ensure a certain electrical decoupling from the soma [40], [57], [59], [64]. The main function of distal, postsynaptic terminals may therefore be to trigger local output rather than to control somatic firing. T-channels could very well play a role in this localized release machinery, as a high distal T-density would increase the duration of the local postsynaptic depolarization (Fig. 7). The possible gain from this would, as we discussed for the Ca^2+^-spikes above, depend on the unknown local condition for dendritic GABA release. It is also possible that there may be multiple trigger mechanisms for local GABA-release, as has been observed, e.g., in granule cells in the olfactory system. There, Ca^2+^ entering through NMDA-receptors may trigger vesicle release in a highly localized fashion, whereas voltage-dependent vesicle release also may occur, but then typically triggered by backpropagating APs [65].

Data on synaptic activation in INs is sparse, but one experiment has shown the somatic EPSC-response to the activation of a single synapse with unknown location (see Fig. 4 in [43]). The experimental EPSC had a duration of about 5 ms, and amplitude of about 50 pA. In additional simulations (results not shown), we found that a distal synapse, no matter how fast and strong, could not produce EPSCs that matched both the amplitude and time course of the experimental EPSCs. This is due to the low-pass filtering properties of the long and thin dendrites, which would tend to broaden the somatic EPSCs. With the synapse model that we used, we found that the experimental EPSCs could only be reproduced if we placed it closer to the soma. For example, with a synaptic weight of 2 nS, and with a uniform T-distribution, we obtained EPSCs that agreed with the experimental recordings (amplitude of 50 pA and duration of about 5ms) when the synapse was placed 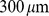 from the soma (branch 1). The EPSCs in the experiments [43] were thus likely due to more proximal input.

### Final remarks

A common conception in biology is that any phenotype, such as the T-distribution, has evolved or been adapted to optimize for a certain biological function. In modelling it is also common to use the reverse argument, i.e., if a theoretical study demonstrates that a specific (hypothetical) T-distribution is optimal for a certain function, it can be considered a prediction that this be the real T-distribution. Our simulations did not converge to any such prediction of the real T-distribution, as we found that different T-distributions were optimal for different IN properties (R1-R5). Since all the properties considered here are of putative importance for different aspects of the INs function within the LGN-circuitry, the real T-distribution is likely to reflect a compromise between the different properties (and thereby between different functions).

There is also a theoretical possibility that the effective T-distribution may vary between INs, or even vary dynamically within a single IN. It is known that some neurons may use activity sensors to dynamically regulate the density of ion channels on their membrane to maintain a stable pattern of activity and to compensate for ongoing, state dependent processes [66]. T-channels modulation by acetylcholine, dopamine and several other transmitter substances has also been demonstrated in several excitable cells [67]. Although response properties of INs are modulated by state dependent input from various regions of the brain [38], [40], [57], [68], there is no experimental evidence that T-channels are subject to dynamic regulation or modulation in INs. However, one could speculate that such regulation or modulation could change the effective distribution of T-channels over time. This could be one potential explanation of the discrepancy between the two experimental studies of the T-distribution in INs [26], [29]. It could also provide a mechanism for the IN to switch between a predominantly dendritic and a predominanly somato-axonal output regime.

The study that we have presented here was motivated by the complex role of IN dendrites within the LGN circuitry. As IN dendrites are long, thin and electrotonically non-compact, and as they serve dual roles as both input and output channels, we hypothesized that the subcellular T-distribution could play a particularly important role in this neuron type. However, although our simulations and motivations were cell specific, we believe that our results shed light on the way the T-distribution affects neural signalling in general.

## Model and Methods

### Simulation

Simulations were run with the NEURON/Python simulating environment [69]. We used a reduced version of the previously developed multicompartmental model of the LGN IN [45], implemented in NEURON [70]. The original model was adapted to current clamp recordings from two different interneurons. It was presented with in two versions (parameterizations P1 and P2), which were able to capture the somewhat different response properties of the two neurons. The reduced model adopted the morphology and the passive properties from the original model [45]. Simulations based on a reduced version of parametrization P2 are presented in the main article. Simulations based on a reduced version of parametrization P1 are found in Figs. S4 - S9. We chose to use P2 for the main presentation. This was because P2 had a more hyperpolarized resting potential (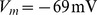) compared to P1 (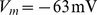), and showed the most pronounced effects of T-channel activation. However, qualitatively similar results were obtained with both parameterizations.

### Morphology

The morphology used in all simulations was based on a realistic, 3D reconstruction of a mouse IN (Fig. 1A). The model interneuron consisted of a soma and 104 dendritic sections, that were subdivided in smaller segments, resulting in a total of 330 segments. The total surface area of the model IN was 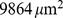; the summed length of all dendrites was 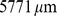; the longest dendrite was 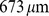, and the mean somatodendritic diameter was about 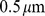.

### Passive properties

The axial (cytoplastic) resistivity, 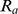, the membrane capacitance, 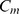 and the membrane resistance, 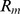 were adopted from the original model [45] for both P1 and P2. The values that were used are indicated in Table 1.

**Table 1:** Parameter sets P1 and P2.

Parameter	Description	P1	P2
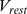	Resting potential	63 mV	-69 mV
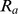	Axial (cytoplastic) resistivity	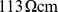	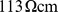
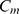	Membrane capacitance	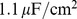	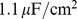
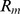	Membrane resistance	22 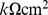	45 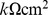
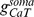	Max. CaT- conductance in soma	1.2e-5 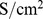	8.5e-6 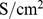
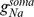	Max. sodium conductance in soma	0.18 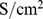	0.18 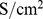
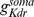	Max. Kdr- conductance in soma	0.34 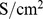	0.4 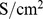
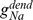	Max. sodium conductance in dendrites	0.0063 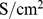	0.0063 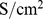
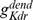	Max. Kdr- conductance in dendrites	0.0051 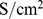	0.006 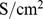
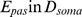	Reversal potential in soma distribution	-65.55 mV	-70.82 mV
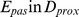	Reversal potential in proximal distribution	-65.45 mV	-70.77 mV
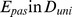	Reversal potential in uniform distribution	-65.17 mV	-70.66 mV
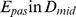	Reversal potential in middle distribution	-65.12 mV	-70.64 mV
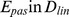	Reversal potential in linear distribution	-65 mV	-70.6 mV
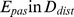	Reversal potential in distal distribution	-65.2mV	-70.72 mV

P2 was used in the main part of the paper. Simulations with P1 can be found in the supporting information.

As T-channels have a nonzero activation level around the resting potential, changing the T-distribution could lead to changes in the resting potential. We prevented this from happening by adjusting the reversal potential of the passive leak current (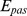) so that the IN always had the same somatic resting potential (-69 mV in the P2 version, and -63 mV in the P1 version).

### Ion channels

Of the seven active ion channels in the original model [45], only three were included in the reduced model. These were, in addition to the T-type Ca^2+^-channel, the traditional AP-generating Na^+^- and delayed-rectifier K^+^-channels (Na and K_dr_). The conductances of 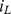, 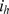, 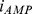 and 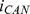 channels were set to 0.

We had several reasons for using a reduced version of the original multicompartmental model [45], excluding some of the ion channels (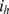, 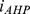, 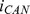 and 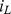 -channels). Firstly, stripping the model down to the essentials allowed us to investigate the effect of the T-distribution in isolation, and made the results easier to interpret. Secondly, the subcellular ion-channel distribution is poorly known for most ion channels in INs. Adding additional mechanisms would have meant introducing additional unconstrained parameters to the model. It is uncertain whether this would have led to more realistic simulations, especially when it comes to dendritic signalling. Thirdly, the fact that the simplified model was sufficient to generate all the essential response features that we were interested in exploring (Fig. 2), validated our conclusions, at least on a qualitative level.

### Na and K_dr_-channels

The Na and K_dr_-channels were described by the standard Hodgkin-Huxley formalism [71]:
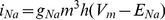
(1)

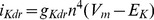
(2)


We adopted the Na^+^ and K^+^ reversal potentials (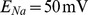 and 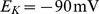), and the kinetics of the gating variables 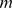, 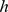 and 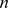 from the original model [45]. However, in the reduced (new) model, we adjusted the Na-activation threshold and the maximum conductances (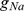 and 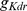) to account for recent experimental studies, which reported that (i) somatically generated APs successfully invade distal dendrites of INs, whereas (ii) synaptic input to distal dendrites was never observed to evoke APs that *initiated* locally in the dendrites [44].

In both P2 and P1, the sodium kinetics which was shifted by -0.2 mV (from -50.3 mV to -50.5 mV) relative to the threshold in the original model. These values were found to (i) ensure successful (back-)propagation of APs into distal dendrites, and (ii) reduce as much as possible the ability of dendritic compartments to evoke local APs.

### T-channels

The model of the T-channel was adopted from the original model [45], but is described in detail here due to its high relevance for the current project. It was modelled using the Goldman-Hodgkin-Katz formulation [72]:
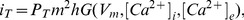
(3)which describes the T-current (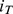) in terms of a maximum permeability for fully open channels (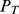 with units cm/s). Like the conductances (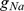 and 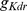), the permeability essentially reflect the density of the respective ion channel type on the cellular membrane. The function
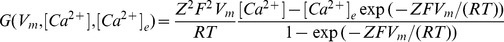
(4)essentially accounts for the dependence of the Ca^2+^-reversal potential on intracellular (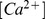) and the extracellular (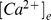) Ca^2+^-concentrations. In Eq. 4, 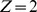 is the valence of the Ca^2+^-ions, 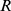 is the gas constant, 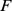 is Faraday's constant and 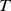 is the absolute temperature. Note that the function 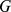 (Eq. 4) has units 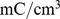, so that the product 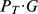 has the units (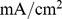) of a current density.

The extracellular Ca^2+^-concentration was assumed to be constant (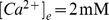). The intracellular Ca^2+^-dynamics was modelled as a leaky integrator:
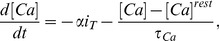
(5)with a 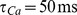), 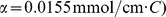 and 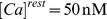 as in the original model [45].

The rationale behind using a different modelling scheme for T-channels is that intracellular Na^+^ and K^+^-concentrations typically are on the orders of several millimolars, and change little during normal neural activity. In contrast, the intracellular Ca^2+^-concentration is on the order of nanomolars and may experience substantial relative changes following Ca^2+^-influx.

The model of the T-channel was adopted from the original model, and we refer to that work for a description of the kinetics of the gating variables 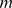 and 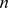. Only the distribution of 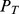 was different from the current work, as described below (note that in the original model [45], 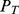 was denoted 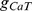 as we mistook it for a conductance).

#### T-channel distribution

The model was presented in six versions (Fig. 1B), characterized by distributions of T-channels over the somatodendritic membrane. The T-channel distributions were normalized so that all model versions had the same mean permeability 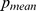 (i.e., the same total number of T-channels) in the neuron as a whole. The normalization criterion was:
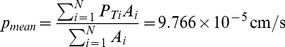
(6)where the sum is taken over all 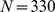 segments in the morphology, so that 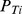 is the permeability in segment 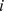 and 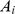 is the surface area of segment 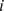. The normalization factor 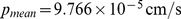 corresponded to the total T-permeability in the original model [45] (also obtained by using Eq. 6). The distributions, i.e., permeability as a function of distance, 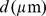, from soma are summarized below:

- soma: All T-channels were concentrated in the soma.
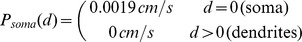

- proximal: Gaussian distribution, mean value, 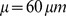, standard deviation 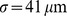.
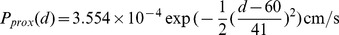

- uniform: All the segments throughout the IN have the same 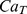 channel density.
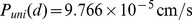

- middle: Gaussian distribution, mean value 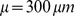, standard deviation 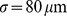.
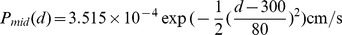

- linear: A density that increases linearly with distance from the soma.
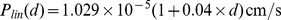

- distal: Gaussian distribution, mean value 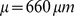, standard deviation 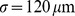.
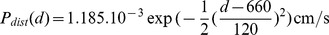

### Synapse model

Synaptic input was modelled using a sum of two exponentials with rise and decay time constants of 0.5 ms and 2 ms, respectively, and with a reversal potential of 10mV. These values have properties that correspond to those found for the AMPA synapse in TC cells [41]. The synaptic weight was varied between 0 and 4 nS.

In the simulations shown in the Results section, the synapse was placed in the distal dendrites. Three different branches were investigated, and synapse locations in branch 1, 2 and 3 had distances 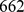, 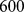 and 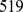 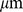 from the soma, respectively.

### Output

In all simulations the model output was the voltage response to a given input. Model output was as specified in the different subsections in the results section and included voltage amplitude, number of action potentials, and calcium spike magnitude. Calcium spikes typically had a duration of 100 - 200 ms and were followed by a small undershoot, i.e., a brief period with 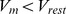. Calcium spike magnitude was calculated as the integral of the function 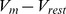 taken from the stimulus onset to the time where the voltage descended below the resting potential.

## Supporting Information

Figure S1
**EPSPs obtained with different T-distributions (with nonzero Na^+^-conductance)**. (**A-D**) EPSPs at different locations along a single dendritic branch (branch 3), as a response to synaptic input (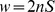) applied to the dendritic endpoint. Different panels represent different T-distributions, as indicated. EPSPs were attenuated upon propagation from the synapse (dark coloured curves) to the soma (light coloured curves). For distributions with a high density of T-channels close to the synapse, synaptic activation evoked local, dendritic Ca^2+^-spikes (C,D).(EPS)

Figure S2
**Magnitude of EPSP for different T-distributions (with nonzero Na^+^-conductance).** (**A–C**) Area of EPSP as a function of distance from soma in 3 different branches (each panel represents a distinct branch). In each branch, synaptic input was applied to the dendritic endpoint. For all distributions, magnitude of EPSP decreased upon propagation towards the soma. (**D–F**) Somatic EPSP magnitude as a function of synaptic weight, 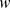. The somatic EPSPs had bigger magnitude for T-distributions with a high density of T-channels close to the synapse. The synaptic weight in **A–C** was 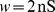. (A-F) Different coloured lines correspond to different T-distributions. Black lines (null distribution) correspond to the case without T-channels.(EPS)

Figure S3
**Impact of morphology of dendritic branches on signal propagation.** (**A**) The distal part of branch 3. In the paper, we only plotted signals propagating along the *main* path, i.e. the direct path from the soma to the most distal dendritic endpoint (dark blue segments). However, the rest of the dendritic tree (light blue segments) also had a significant impact on the propagation of the signal. (**B**) Waveform of backpropagating APs at different distances from soma along the main path (blue curves) and highlighted in red, green and black at selected locations, marked in **A** by arrows of the corresponding colour. APs that propagate along the main path increase in amplitude upon approaching a nearby endpoint. The red curve represents a signal in a neighbouring segment (not along the main path), which also happens to be at an endpoint. The light blue curves directly beneath the red curve in **B**, show the AP waveforms in the region marked with an ellipse in **A**, situated at around 200–250 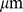 from the soma. Specific morphological features like this explain the attenuation profiles shown in the main part of the article (Fig. 4). APs may also take a deflection at branch endpoints and propagate back in the direction of the soma. At the location marked with a green arrow in **A**, we therefore first observe the primary AP from the soma, and then the secondary AP propagating towards the soma (green curve in **B**). In this specific case, the secondary AP has a higher amplitude than the primary AP. Such deflected, secondary APs explain features observed for e.g. in Fig. 4F in the main article, where the AP peak amplitude was observed earlier in the branch endpoints than at distal dendritic locations close to the endpoint.(EPS)

Figure S4
**P1 - Response to somatic current injection.** Analogous figure to Fig. 2 in main text, but with parameterization P1. (**A**) A prolonged (1000 ms) stimulus protocol, 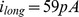 (A-iv), evoked an initial high AP-firing frequency phase as in P2. With P1 however, the subsequent AP firing showed some irregularity that was not observed with P2 (e.g. for the soma and distal distributions). This irregularity was related to Ca^2+^-oscillations evoked by the generally higher T-density in P1 compared with P2. (**B**) A brief (10 ms) stimulus protocol, 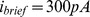 (B-iv), evoked a burst of APs. When AP firing was suppressed by setting the Na^+^-conductance to 0, the Ca^2+^-spike underlying the burst was revealed (dashed lines). (**C**) Number of regular APs elicited during the last 700 ms of the stimulus period as a function of prolonged stimulus amplitude (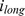) for all T-distributions. With P1, stronger stimulus amplitude (compared to P2) was required to get firing in the last 700 ms of the stimulus. However, the same patterns can be observed. (**D**) Number of APs elicited in bursts as a function of brief stimulus amplitude (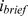) for all T-distributions.(EPS)

Figure S5
**P1 - Effect of T-distribution on AP backpropagation.** Analogous figure to Fig. 4 in main text, but with parameterization P1. (**A–C**) In each segment of a selected branch, the peak amplitude of last action potential (Fig. S4A) (left panel) and (**D–F**) the time taken from soma to reach peak amplitude (right panel) are plotted as a function of distance from soma (peak time, 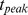, was plotted relative to the time of the somatic AP-peak (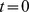)). The study of AP propagation was more complicated in P1 than it was in P2. As implied by the irregularity in the AP-firing pattern (Fig. S4), two successive APs did not experience identical local conditions. In most cases, AP propagation still followed the same general trend as we obtained with P2 (see main article). An exception was found in branch 3, where 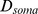 and 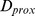 unexpectedly gave rise to faster AP-propagation than the uniform distribution (**F**). A closer investigation revealed that this was related to interaction between successive APs, which occurred with 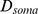 and 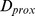 due to the short intraspike intervals (high AP-firing frequency) obtained with these distributions. With these distributions, a subset of the APs failed to successfully invade the distal dendrite. These *unsuccessful* APs experienced passive attenuation in the distal regions of the dendrites. As the membrane was not repolarized in those cases by the fast K^+^ current that accompanies full APs, the depolarizations evoked were more enduring. The membrane potential is thus higher upon arrival of the subsequent AP, which is then able to successfully invade the distal dendrite. This may explain why the APs that did invade the distal dendrites in the case of 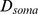 and 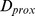 (those plotted in **C** and **F**) were faster than one of the other distributions (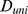), where all APs invaded the distal dendrites. Still, in P1 as in P2, we obtained the fastest AP-propagation with a high distal T-density (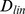 and 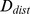). The insight gained from the special case of branch 3, can indicate that our conclusions regarding AP propagation are valid in case of regular AP firing at relatively low firing frequencies (i.e., with relatively long intraspike intervals that prevent interaction between two successive APs).(EPS)

Figure S6
**P1 - Ca^2+^-spikes in the soma for all T-distributions.** Analogous figure to Fig. 5 in main text, but with parameterization P1. (**A–C**) Ca^2+^-spikes, evoked by 10 ms somatic current injections are illustrated here for stimulus amplitudes, 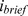, of 100, 150 and 250 pA. AP firing was suppressed by setting the Na^+^-conductance to 0. Overall, while similar trends as in P2 were observed, the calcium spikes observed with parameterization P1 are less pronounced than in P2. (**D**) Response magnitude of Ca^2+^-spikes as a function of the stimulus amplitude for all T-distributions. With P1, regardless of the stimulus, the Ca^2+^-spikes response in the soma are always strongest in the soma and proximal distributions.(EPS)

Figure S7
**P1 - Ca^2+^-spikes in the dendritic tree.** Analogous figure to Fig. 6 in main text, but with parameterization P1. (**A–C**) Magnitude of Ca^2+^-spikes as a function of distance to the soma. The Ca^2+^-spike magnitude clearly reflected the underlying T-distribution. Ca^2+^-spikes were evoked by brief (10 ms) current injections with stimulus amplitude (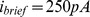). (**D-F**) Magnitude of Ca^2+^-spikes at dendritic endpoints as a function of stimulus amplitude 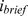, illustrated for three different dendritic branches. The response magnitude was always biggest for T-distributions with a high distal T-density.(EPS)

Figure S8
**P1 - EPSPs obtained with different T-distributions.** Analogous figure to Fig. 7 in main text, but with parameterization P1. (**A–D**) EPSPs at different locations along a single dendritic branch (branch 3), as a response to synaptic input (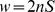) applied to the dendritic endpoint. Different panels represent different T-distributions, as indicated. EPSPs were attenuated upon propagation from the synapse (dark coloured curves) to the soma (light coloured curves). The Na^+^-conductance was set to 0 to suppress the dendritic Na^+^-spikes that would have been evoked in distributions with a high density of T-channels close to the synapse.(EPS)

Figure S9
**P1 - EPSP amplitudes for different T-distributions.** Analogous figure to Fig. 8 in main text, but with parameterization P1. (**A–C**) EPSP amplitude (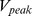) as a function of distance from soma in 3 different branches. In each branch, synaptic input (weight =  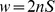) was applied to the dendritic endpoint. The attenuation profiles were fairly similar for all distributions except for 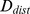 which experienced a boost. This effect was more pronounced in branches 1 and 3 than in branch 2, due to morphological differences between the branches. Branch 2 has fewer ramifications (i.e. a smaller membrane area) and thus fewer T-channels in the distal regions than branches 1 and 3. (**D–F**) Somatic EPSP amplitude as a function of synaptic weight, 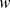. The somatic EPSPs had bigger amplitude for T-distributions with a high density of T-channels close to the synapse.(EPS)
